# Psychosocial determinants of HIV testing across stages of change in Spanish population: a cross-sectional national survey

**DOI:** 10.1186/s12889-017-4148-4

**Published:** 2017-03-07

**Authors:** Maria Jose Fuster-RuizdeApodaca, Ana Laguia, Fernando Molero, Javier Toledo, Arantxa Arrillaga, Angeles Jaen

**Affiliations:** 1 Spanish Interdisciplinary AIDS Society (Sociedad Española Interdisciplinaria del Sida, SEISIDA), C/Doctor Fleming 3, 28036 Madrid, Spain; 2 Universidad Nacional de Educación a Distancia (UNED), Facultad de Psicología, C/Juan del Rosal, 28040 Madrid, Spain; 3 Plan de VIH/Sida Dirección General de Salud Pública, Gobierno de Aragón. Vía Universitas 36, 5a Planta, 50017 Zaragoza, Spain; 4 Plan del Sida e Infecciones de Transmisión Sexual (AIDS Strategy), Osakidetza-Servicio vasco de salud, Avda. Navarra, 14, 20013 San Sebastián, Spain; 5 Fundació Docència i Recerca Mútua Terrassa, C/Sant Antoni n° 19, 08221 Terrassa Barcelona, Spain

**Keywords:** HIV, Health behavior, HIV-testing, Late diagnosis

## Abstract

**Background:**

The goal of this research is to study the psychosocial determinants of HIV-testing as a function of the decision or change stage concerning this health behavior. The determinants considered in the major ongoing health models and the stages contemplated in the Precaution Adoption Process Model are analysed.

**Methods:**

A cross-sectional survey was administered to 1,554 people over 16 years of age living in Spain by a computer-assisted telephone interview (CATI). The sample design was randomised, with quotas of sex and age. The survey measured various psychosocial determinants of health behaviors considered in the main cognitive theories, the interviewees' stage of change concerning HIV-testing (lack of awareness, decision not to act, decision to act, action, maintenance, and abandonment), and the signal for the action of getting tested or the perceived barriers to being tested.

**Results:**

Approximately two thirds of the population had not ever had the HIV test. The predominant stage was lack of awareness. The most frequently perceived barriers to testing were related to the health system and to the stigma. We also found that the psychosocial determinants studied differed depending on the respondents' stage of change. Perception of risk, perceived self-efficacy, proximity to people who had been tested, perceived benefits of knowing the diagnosis, and a positive instrumental and emotional attitude were positively associated with the decision and maintenance of testing behavior. However, unrealistic underestimation of the risk of HIV infection, stereotypes about the infection, and the perceived severity of HIV were associated with the decision not to be tested.

**Conclusions:**

There are various sociocognitive and motivational profiles depending on people’s decision stage concerning HIV-testing. Knowing this profile may allow us to design interventions to influence the psychosocial determinants that characterise each stage of change.

**Electronic supplementary material:**

The online version of this article (doi:10.1186/s12889-017-4148-4) contains supplementary material, which is available to authorized users.

## Background

Late diagnosis of HIV infection is a major problem. Late diagnosis refers to people with HIV with a CD4 count below 350 cells/ul, or having an AIDS-defining event regardless of the CD4 count [1]. In Spain, it is estimated that at least 30% of people with HIV do not know that they are infected [2]. Although the pattern in Spain consists of infection mainly concentrated in men who have sex with men (MSM), higher percentages of late diagnoses are found in heterosexuals [3]. Late diagnosis has three main consequences. Firstly, those people unaware of their HIV-positive diagnosis cannot benefit from treatment, which increases their risk both of morbidity and death [4]. Secondly, the later the diagnosis, the higher the cost of treatment and care [5]. Lastly, people diagnosed late can infect other people. Research provides data indicating that between 54% and 65% of new infections are caused by people who were unaware that they were infected [6].

HIV-testing is a health behavior that can be influenced by various psychosocial determinants. There are many models of health and psychological theories that explain the factors that determine or influence a person to adopt or reject a health behavior. Theories are typically categorised into (1) continuum models and (2) stage models.

Continuum models describe predictors that are combined into a linear prediction equation that places individuals along a continuum of behavior likelihood. If one or more of these determinants are strengthened, the likelihood of behavior or behavior change increases [7]. Among these continuum models, a group of theories focuses on cognitive variables as a part of behavioural change. These models share the assumption that attitudes and beliefs are important determinants of health behavior [8, 9]. This perspective includes theories such as the Health Belief Model (HBM) [10, 11], the Protection Motivation Theory (PMT) [12], the Social Cognitive Theory (SCT) [13], or the Theory of Reasoned Action or Planned Behavior (TRA, TPB) [14, 15]. These theories include, among others, the following psychosocial determinants that are relevant for the purpose of this research: (a) the perception of personal vulnerability to the health problem; (b) the perception of threat posed by the disease, both by the risk to health and, in the case of HIV, by the threat of stigma [16]; (c) the assessment of one's coping, that is, of the behavioural alternatives that may reduce the threat and the expectation of the efficacy of one's response; (d) the belief in one's personal capacity or self-efficacy to successfully perform the health behavior; (e) the expectation of behavioural outcomes, that is, the positive or negative effects produced, the appraisal of the outcomes of performing the health behavior; (f) attitude, which is determined by beliefs and the appraisal of the outcomes of the behavior; (g) the subjective norm, which consists of beliefs about referents’ opinions and the motivation to comply with those referents; and (h) signals for taking action.

Stage models are based on the assumption that behavior change takes place at several discrete stages. People will be more or less willing to perform a health behavior depending on their current stage within the change process. One of those models is the Model of the Precaution Adoption Process (MPAP) [16, 17], which considers several stages in the recognition, adoption, and maintenance of health behavior. The core of this model is a sequence of six stages: “unaware of the issue”, “aware of the issue but not personally engaged”, “engaged and deciding what to do”, “planning to act but not yet having acted”, “acting”, and “maintenance”. There is an additional stage if the conclusion of the decision-making stage is not to act, but this is not a stage along the route of action [17]. The existing research findings suggest that diverse psychosocial determinants differentially influence health behavior depending on people's stage of change [7, 18] and that the study of these stages can help to focus interventions on the specific barriers that prevent going from one stage to the next [19]. However, few studies have examined the integration of the psychosocial determinants contemplated in the continuum health models and the stage models [20] and, to our knowledge, in the case of the late diagnosis of HIV, there are no studies.

In addition, in the literature on late diagnosis of HIV, most of the studies that analyse barriers to diagnosis and their correlates focus on sociodemographic factors, and their main limitation is their inadequate approach to the research of psychosocial predictors [21]. Furthermore, few investigations study these psychosocial factors within the context of the theoretical frameworks of health behaviors [21, 22]. However, knowing these aspects is essential to provide an effective response to this major public health problem. Therefore, the goal of this research is to study the psychosocial determinants of HIV-testing in a representative sample of the Spanish population as a function of their stage of change about being tested. We used the concepts and determinants contemplated in the above-mentioned health models (HBM, PMT, SCT, TRA) and considered in the MPAP stages.

## Methods

### Design and procedure

A cross-sectional survey was administered to 1,554 people over 16 years of age residing in Spain (Additional file 1). The sample design was randomised, with quotas of sex and age. To ensure adequate dispersion, we started with an initial distribution of the interviews in proportion to the population by Nielsen areas. To select the homes, we assigned original telephone numbers to each Nielsen area in proportion to its population. The people in each home were selected randomly, applying quotas of sex and age. The margin of error of the survey was of ± 2.5%, with a 95.5% degree of confidence in a setting of maximum dispersion (*p* = *q* = 50). The data were collected in June of 2012.

A computer-assisted telephone interview (CATI)^1^ was conducted. We required 17,647 telephone contacts. The response rate was 8.8%, that is, we had to perform 11.4 calls for each completed interview. Mean interview duration was 11 min. All the processes of the study were carried out according to the A50/000005 Market and Opinion Research System, following the ISO Norm 20252, certified by AENOR and with the Behavior Code of ESOMAR.

### Instrument

To develop the survey, the following procedure was conducted. Firstly, a literature review on the topic under study was performed. Secondly, a qualitative study was conducted in which 25 people with late HIV were interviewed [16]. The third step included drafting a pool of potential items covering the variables under study. For this purpose, the research team agreed on and wrote a definition of the constructs to be evaluated, drafted or adapted items from the reviewed measures tapping the defined construct, and rated the items’ clarity, relevance, and representativeness. Thirty-five items were selected from an initial pool of 54 items. Finally, 15 telephone interviews were performed to ensure comprehension and feasibility of the length of the survey.

The survey contained a first group of items (*n =* 18) of *psychosocial determinants of health behaviors* considered by the models cited in the introduction (HBM, PMT, SCT, TRA, TPB) [16, 22–29]. The following determinants were assessed: perceived HIV risk, underestimation of risk of HIV infection, invulnerability associated with stereotypes, perceived threat, perceived severity, perceived stigma, self-efficacy to undergo testing and to deal with a positive test outcome, social proximity to HIV-tested people, motivation to be tested if so requested by reference people, perceived benefits of being tested, and attitude (instrumental and emotional dimensions). One additional item was included to explore the opinion about the likelihood of being infected by HIV through sexual intercourse when condoms are not used. The items can be found in Table 1.

**Table 1:** Psychosocial determinants, items used in the survey to measure each determinant, and response percentages in the total sample surveyed

Psychosocial determinant	Item summarised	Percent	95% CI	Variance	Skewness	Kurtosis
Perceived risk	Thinks that he/she could become infected with HIV^1^	9.2ª	[7.7, 10.7]	0.47	1.01	2.34
Underestimation of risk of infection (unrealistic optimism)	Thinks he/she is less likely to become infected by HIV than the average of the population^1^	53.8^b^	[51.3,56.3]	1.01	0.18	-1.01
Knowledge about HIV sexual transmission	Considers that it is likely to become infected by HIV through sexual intercourse if condoms are not used	90.5ª	[89, 92]	0.45	-0.76	0.15
Invulnerability associated with stereotypes	Does not think he/she can become infected because only certain groups of people have HIV^1^	41.7^b^	[39.2,44.2]	0.87	0.52	-0.84
Perceived threat	Would feel afraid if he/she received a positive diagnosis^1^	81.9^b^	[79.2, 83.2]	0.41	-0.65	-0.53
Perceived severity	AIDS is a deadly disease^1^	39^b^	[36.5,41.5]	0.92	0.51	-0.85
	AIDS is a very serious disease^1^	81.8^b^	[79.8, 83.8]	0.55	-0.48	-0.33
Perceived stigma	People with HIV are highly rejected in society^1^	78.9^b^	[76.8,81]	0.69	-0.55	-0.25
Self-efficacy	Perceived ability to be tested^1^	87.9^c^	[86.2, 89.6]	0.43	-1.67	2.72
	Perceived ability to deal with a positive result^1^	76.4^c^	[74.2, 78.6]	0.70	-0.78	0.22
Social proximity	Has people nearby who have been tested^1^	9.4^d^	[7.9, 10.9]	0.60	0.97	0.53
Motivation to comply with referents	Would be tested if asked to by reference persons^1^	95.7^b^	[94.7, 96.7]	0.24	-2.63	6.29
Perceived benefits of knowing a positive diagnosis	Receiving treatment as soon as possible and controlling the disease^1^	99^b^	[98.5, 99.5]	0.02	-5.95	34.3
	Protecting the partner and preventing transmission^1^	99.1^b^	[98.6, 99.6]	0.02	-5.95	34.3
	Having information about the diagnosis would relieve you about your health status^1^	94.3^b^	[93.1, 95.5]	0.09	-2.57	4.76
Instrumental attitude (*M* ± *SD*)	Considers it harmful-beneficial to be tested^2^	8.14 ± 2.56		3.51	-2.24	6.25
	Considers it useless-useful to be tested^2^	8.52 ± 2.56		1.34	-2.27	4.49
Emotional attitude (M ± SD)	Considers it unpleasant-pleasant to be tested^2^	4.44 ± 3.63		12.48	-0.12	-1.14
	Considers it stressful-relaxing to be tested^2^	4.02 ± 3.57		11.84	0.38	-0.94

Data provided in percentages, except where specified. *CI* confidence interval. *N* number of items. ^1^Items have a 4-point response range. ^2^Items have a range of 10 points

^a^Percentage of people responding “fairly likely”/“very likely”. ^b^ Percentage of people responding “agree somewhat” or “totally agree”. ^c^ Percentage of people responding “fairly capable” or “very capable”. ^d^ Percentage of people responding “quite a lot” or “many”

In addition, the survey contained a question to determine at which *stage of change with regard to HIV-testing* the respondents were. The response categories were adapted to the stages considered in the MPAP [17] and were as follows: (a) Lack of awareness: has never been tested and has never thought about it; (b) Decision not to act: has not been tested and has decided not to be tested; (c) Decision to act: has not yet been tested but intends to be tested soon; (d) Action: has been tested at some time; (e) Maintenance: is tested regularly; and (f) Abandonment: has been tested but will not be tested any more.

The survey also contained a few specific items depending on whether or not the interviewees had been tested. People who had been tested were asked what had led them to testing, that is, the *signal for taking action*. The items (*n =* 7) were drawn from a survey by the Kaiser Family Foundation and the qualitative study that preceded this population-based study [16, 29]. Moreover, people who had not been tested and those who had decided to quit testing were asked about the *perceived barriers to testing*. They were asked about structural barriers (*n =* 3) and barriers related to the health system (*n =* 3) and to stigma (*n =* 3) [16, 29, 30]. These items and the response options can be found in Table 3.

### Data analysis

First, exploratory analysis was performed, and the verification of the multivariate analysis assumptions was checked. Results showed that the items did not match the univariate normal distribution. Values of variance, skewness, and kurtosis can be seen in Table 1.

Next, to simplify the data analysis, when a variable was composed of more than one item, and the items were adequately correlated, they were averaged to yield a composite score. We created a composite score with the following variables: perceived benefits of knowing a positive diagnosis (*n =* 3, *α* = .70), instrumental attitude (*n =* 2, α = .70, *r* = 55), and emotional attitude (*n =* 2, α = .72, *r* = 56).

Finally, to analyse the differences in psychosocial determinants and sociodemographic characteristics as a function of the participants' stage of change, we used one-factor ANOVA for continuous variables and Chi-square for categorical variables. Tukey’s HSD test was performed to compare differences among groups. Although ANOVA is robust about the violations of normality and homoscedasticity criteria [31, 32], the results were also checked with a non-parametric technique (Kruskal-Wallis).

## Results

### Participants

Of the people contacted, 3.6% did not know what AIDS was, so they did not complete the interview, leaving a valid sample of 1,499 interviews. The characteristics of the participants are shown in Table 2.

**Table 2:** Demographic characteristics of the total sample and of the people who made up each stage of change

			Stage of change					
Characteristics		Total	Lack of awareness	Decision not to act	Decision to act	Action	Maintenance	Abandonment
*N*		1499	802	204	67	291	74	38
Sex	Male	47.6	49.1	50.5	50.7	42.6	45.9	39.5
	Female	52.4	50.9	49.5	49.3	57.4	54.1	60.5
Age	*M* ± *SD*	45.34 ± 16.94	45.61 ± 18	54.25 ± 17.9	32 ± 13.8	41.63 ± 11.5	44.1 ± 11.8	45 ± 12
Nationality	Spanish	95.5	97	95.6	82.1	94.2	95.9	94.7
	Other European country	1.1	1.1	1.5	4.5	0.7	0	0
	Latin American	2.7	1.4	1	13.4	5.2	4	2.6
	Other	0.7	0.5	2	0	0	0	2.6
Level of education	No education	2.8	3.5	3.9	1.5	1.4	0	2.6
	Primary education	20.9	21.6	32.8	20.9	15.1	12.2	10.5
	Secondary education	37.2	38.2	31.9	49.3	35.4	33.8	47.4
	Middle university education	12.9	12	10.8	10.4	15.1	20.3	15.8
	Higher university education	21.3	19.6	16.7	13.4	28.2	28.4	18.4
	Other	4.9	5.2	4	4.5	4.8	5.4	5.3
Habitat Quota	Less than 20,000	30	32.4	29.9	28.4	25.8	24.3	31.6
	From 20,001 to 50,000	13.3	12.8	14.7	9	14.8	17.6	10.5
	From 50,001 to 200,000	23.1	23.2	25.5	32.8	20.6	16.2	23.7
	From 200,001 to 500,000	14.9	15.1	12.7	6	16.2	20.3	15.8
	More than 500,000	18.6	16.5	17.2	23.9	22.7	21.6	18.4
Socioeconomic status	High	2.9	2.5	2.5	4.5	3.8	2.7	2.6
	Medium-high	23.7	23.7	25	11.9	24.7	29.7	15.8
	Medium	23.3	21.6	17.6	25.4	29.9	28.4	26.3
	Medium-low	44.7	47.5	51	52.2	33.7	33.8	52.6
	Low	5.3	4.7	3.9	6	7.9	5.4	2.6
Employment situation	Working	39.5	35.1	28	31.4	56	48.7	55.3
	Unemployed	13.9	14.6	7.8	14.9	15.8	17.6	13.2
	Retirees and pensioners	20	21.7	36.3	6	10.7	9.5	18.4
	Students	10.3	12.7	7.8	32.8	3.4	4.1	0
	Other (home-makers/others)	16.3	15.9	20.1	14.9	14.1	20.3	13.2

Data provided in percentages, except where specified

Abbreviations: *SD* standard deviation

#### Situation of the Spanish population concerning HIV-testing: stages of change, signal for action, and perceived barriers

Of the interviewees, 53.5% were at the stage of lack of awareness, 13.6% at the stage of deciding not to act, 4.5% at the stage of deciding to act, 19.4% in the action stage, 4.9% in the maintenance stage, 2.5% in the abandonment stage, and 1.5% did not answer the question.

People who had been tested in the past and people who stated that they intended to be tested soon were asked about the reasons that led them to adopt this health behavior (*N =* 470). The most frequently mentioned reasons were that it seemed a good idea, the fact that it was just one more test included in a general health check, and the recommendation of a healthcare professional (Table 3).

**Table 3:** Signal for taking action and perceived barriers to testing

	Percent	95% CI
Reasons to be tested (signal for action)^1,a^		
It just seemed a good idea	56.4	[51.9, 60.9]
The doctor or other health professional suggested getting tested	33.2	[28.9, 37.5]
Is worried about the possibility of being infected	16.4	[13.1, 19.7]
The partner or someone important has suggested getting tested or has asked whether he/she has been tested	9.6	[6.9, 12.3]
Felt ill and decided, or has decided, to get a general checkup	19.6	[16, 23.2]
Had doubts about whether a partner could have HIV or any other sexually transmitted infection	12.3	[9.3, 15.3]
The doctor or nurse told him/her that they had been tested because it was just one more test of the health checkup	51.1	[46.6, 55.6]
Does not know/Does not respond	4	[2.2, 5.8]
Perceived barriers to testing^2,b^		
Related to the health system		
The doctor has not recommended taking the test	81.4	[79.1–83.7]
Would feel ashamed to talk about the test with the doctor	12.2	[10.3–14.1]
Worried about what the doctor might think if he/she requests getting tested	9.3	[7.6–11]
Related stigma		
Concerned that someone may expose him/her	25.2	[22.6–27.8]
Is afraid his/her name might appear in public records	29.3	[26.6–32]
Concerned that loved ones will reject him/her	42.9	[40–45.8]
Structural barriers		
Has no time to go to take the test	13.6	[11.6–15.6]
Does not know where to go to take the test	40.1	[37.2–43]
The place where the test is done is far away	7.47	[5.8–9]

*CI* Confidence interval

^1^ *N =* 470 (includes people at the stages of deciding to act, action, and abandonment)

^2^ *N =* 1044 (includes people at the stages of lack of awareness, deciding not to act, and abandonment)

^a^Items have a dichotomous response. Percentage of people who respond “Yes.” ^b^ Items have a 4-point response range. Percentage or people who state that they agree “pretty much” or they “strongly agree”

Moreover, we explored the possible barriers to HIV-testing among people who had not been tested — stage of lack of awareness and stage of deciding not to act—as well as in people who had decided not to repeat the test (abandonment stage).

As shown in Table 3, the barrier most frequently mentioned was the lack of the doctor's recommendation. In addition, nearly 43% of the people interviewed expressed concern about rejection by their loved ones, and more than a fourth feared that someone might expose them or that their names might appear in public records. Among the structural barriers, the most frequently mentioned was the lack of knowledge about where the test can be done.

### Psychosocial determinants associated with HIV-testing

As shown in Table 1, a very low percentage of the population perceived themselves as being at risk of HIV. In addition, about half of the respondents thought that they were less likely to become infected than the average population, and a little more than 40% thought that their low perception of risk was associated with the belief that AIDS is an infection that affects certain groups of people. However, more than 90% considered that it was fairly likely or very likely that one would become infected if condoms were not used during sexual intercourse. Percentages of about 80% feared receiving a positive diagnosis, the perceived seriousness of the disease, and the perceived stigma. Moreover, percentages near or higher than 90% perceived self-efficacy to undergo testing, were motivated to be tested if so requested by reference people and perceived benefits to be tested. However, less than 10% of the interviewed population had social proximity to HIV-tested people.

### Characterisation of the stages of change

**Fig. 1:**
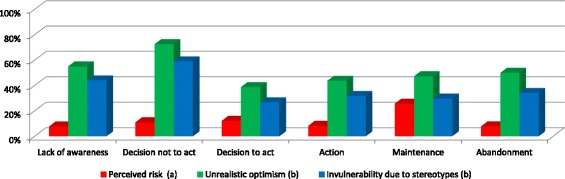
Response percentages of the determinants related to perceived risk of HIV infection across the stages of change. Note: ^a^Percentage of people who answered “fairly likely”/“very likely”. ^b^Percentage of people who answered “agree somewhat” or “totally agree”

**Fig. 2:**
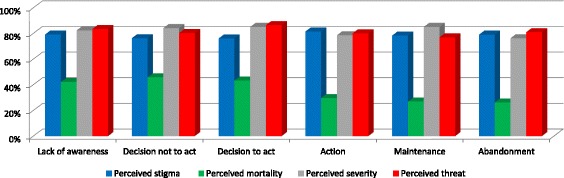
Response percentages in the determinants related to perceived threat across the stages of change. Note: Percentage of people who answered "agree somewhat" or "totally agree”

**Fig. 3:**
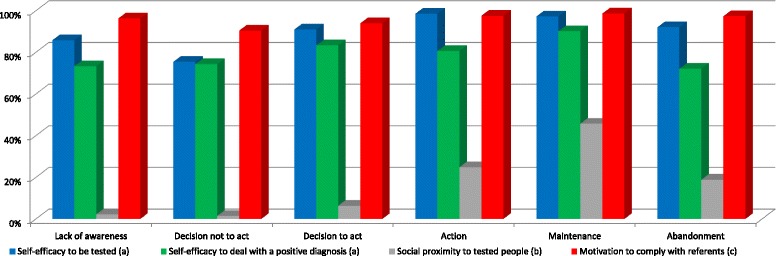
Response percentages of the determinants related to perceived self-efficacy and subjective norm. Note: ^a^ Percentage of people who answered responding "fairly capable" or "very capable”. ^b^ Percentage of people responding "pretty many” or "many”. ^c^ Percentage of people who answered “agree somewhat” or “totally agree”

We found differences in the psychosocial determinants studied according to the different stages of change. The ANOVA results are shown in Table 4. For greater clarity, we show the response percentages of each of these determinants in Figs. 1, 2, and 3. The psychosocial determinants that showed differences at each stage can be seen in Table 5.

**Table 4:** Differences in psychosocial determinants according to stage of change

Psychosocial determinants	Stages of change						
	Lack of awareness	Decision not to act	Decision to act	Action	Maintenance	Abandonment	*F*(*df*)
	M ± SD (%)ª						
Perceived risk^a^	1.67 ± .70	1.69 ± .77	1.94 ± .74	1.73 ± .65	2.05 ± .86	1.72 ± .71	5.43 (5, 1463)***
Underestimation of risk of HIV infection ^a^	2.58 ± 1.07	2.97 ± 1.07	2.22 ± 1.08	2.39 ± 1.1	2.42 ± 1.13	2.55 ± 1.10	8.66 (5, 1457)***
Invulnerability due to stereotypes^a^	2.30 ± 1.13	2.74 ± 1.14	1.87 ± 1.04	1.98 ± 1.08	1.91 ± 1.16	2.08 ± .96	14.9 (5, 1464)***
Perceived threat^a^	3.24 ± .94	3.16 ± .94	3.31 ± .92	3.20 ± 1	3.05 ± 1.08	3.19 ± .99	.80 (5, 1462)
Perceived severity^a^	3.19 ± .83	3.19 ± .76	3.28 ± .75	3.13 ± .83	3.24 ± .77	3.08 ± .81	.65 (5, 1463)
Perceived mortality^a^	2.26 ± 1.03	2.34 ± 1.12	2.28 ± 1.08	2 ± .95	1.96 ± 1.01	1.92 ± 1.02	4.89 (5, 1463) ***
Perceived stigma^a^	3.11 ± .84	2.94 ± .85	3.03 ± .87	3.21 ± .83	3.09 ± .72	3.10 ± .82	2.62 (5, 1466)*
Self-efficacy to be tested^a^	3.39 ± .89	3.10 ± 1.12	3.43 ± .78	3.83 ± .44	3.91 ± .44	3.74 ± .60	25.21 (5, 1458)
Self-efficacy to deal with a positive diagnosis^a^	2.97 ± .85	2.96 ± .94	3.14 ± .87	3.11 ± .78	3.32 ± .77	2.92 ± .84	3.53 (5, 1418)**
Social proximity to tested people^a^	1.23 ± .47	1.15 ± .40	1.59 ± .66	2.02 ± .86	2.36 ± .99	1.73 ± .90	108.78 (5, 1406)***
Motivation to comply with referents^a^	3.76 ± .57	3.56 ± .81	3.72 ± .62	3.84 ± .48	3.92 ± .32	3.89 ± .38	7.24 (5, 1461)***
Benefits of the test^b^	3.81 ± .34	3.67 ± .52	3.90 ± .25	3.87 ± .33	3.91 ± .22	3.93 ± .20	10.37(5, 1470)***
Instrumental attitude^c^	8.11 ± 2.28	7.64 ± 2.81	8.70 ± 1.63	8.96 ± 1.81	8.91 ± 2.06	9.26 ± 1.86	12.54 (5, 1466)***
Emotional attitude^c^	3.98 ± 3.14	3.77 ± 3.47	5.05 ± 3.05	4.80 ± 3.01	5.01 ± 3.47	4.39 ± 3.05	5.51 (5, 1460)***

* *p* < .05; ** *p* < .01; *** *p* < .001

^a^The item has a range of 4 points.^b^The scale has a range of 4 points; α = .70. ^c^The scale has a range of 10 points

**Table 5:** Significant differences among the stages of change and the psychosocial testing determinants

	Decision not to act	Decision to act	Action	Maintenance	Abandonment
Lack of awareness	+ Underestimation of HIV risk***	+ Perceived HIV risk*	- Stereotypes***	+ Perceived HIV risk***	+ Social proximity test***
	+ Stereotypes***	- Stereotypes*	- Perceived mortality**	- Stereotypes*	+ Instrumental attitude*
	+ Perceived mortality	+ Social proximity test***	+ Social proximity test***	+ Social proximity test***	
	- Self-efficacy test***		+ Self-efficacy test***	+ Self-efficacy test***	
	- Motivation referents***		+ Instrumental attitude***	+ Self-efficacy diagnosis*	
	- Benefits of test***		+ Emotional attitude**	+ Social proximity test	
				+ Instrumental attitude*	
Decision not to act		- Underestimation of HIV risk***	- Underestimation of HIV risk***	+ Perceived HIV risk**	- Stereotypes**
		- Stereotypes***	- Stereotypes***	- Underestimation of HIV risk**	+ Self-efficacy test***
		+ Self-efficacy test*	- Perceived mortality	- Stereotypes***	+ Social proximity test***
		+ Social proximity test***	+ Perceived stigma**	+ Self-efficacy test***	+ Motivation referents*
		+ Benefits test***	+ Self-efficacy test***	+ Self-efficacy diagnosis*	+ Benefits test**
		+ Instrumental attitude**	+ Social proximity test***	+ Social proximity test***	+ Instrumental attitude**
		+ Emotional attitude*	+ Motivation referents***	+ Motivation referents***	
			+ Benefits test***	+ Benefits test***	
			+ Instrumental attitude***	+ Instrumental attitude***	
			+ Emotional attitude**	+ Emotional attitude*	
Decision to act			+ Self-efficacy test**	+ Self-efficacy test**	
			+ Social proximity test***	+ Social proximity test***	
Action				+ Perceived HIV risk**	
				+ Social proximity test***	
Maintenance					- Social proximity test***

HSD Tukey: *p < .05; ** *p* < .01; *** *p* < .001. + The score in the variable is higher at the stage in the column than at the stage in the row. - The score in the variable is lower at the stage in the column than at the stage in the row

Stereotypes: invulnerability associated with stereotypes. Self-efficacy test: self-efficacy to undergo testing; Self-efficacy diagnosis: self-efficacy to deal with a positive HIV diagnosis. Motivation referents: motivation to be tested if so requested by reference people. Social proximity test: social proximity to HIV-tested people. Benefits test: perceived benefits of being tested

Perceived HIV risk was higher at the stages of decision to act and maintenance compared to the stage of lack of awareness. It was also higher at the maintenance stage compared to the stages of decision not to act and action.

Underestimation of the risk of HIV infection was lower at the stages of lack of awareness, decision to act, action, and maintenance than at the stage of decision not to act.

The belief that lower perceived risk was due to the fact that they did not consider themselves as belonging to a group at risk of infection (invulnerability due to stereotypes) was lower both at the stage of decision to act and the stage of maintenance than at the stage of lack of awareness. It was also lower at the stage of decision not to act than at the other stages.

Perceived HIV mortality was higher at the stage of decision not to act than at the stage of lack of awareness. On the contrary, perceived mortality was lower at the stage of action than at both the stages of lack of awareness and decision not to act.

Self-efficacy to undergo HIV-testing was lower at the stage of decision not to act than at the rest of the stages. However, it was higher at the stages of action and maintenance than at the stage of decision to act.

Self-efficacy to deal with a positive diagnosis was higher at the stage of maintenance than at both the stage of lack of awareness and of the decision not to act.

Social proximity to tested people was more frequent at the stages of decision to act, action, maintenance, and abandonment than at both the stages of lack of awareness and decision not to act. It was also higher at both the stages of action and maintenance than at the stage of decision to act. However, social proximity to tested people was lower at the stage of abandonment than at the stage of maintenance.

The motivation to be tested if so requested by reference people was lower at the stage of decision not to act than at the stages of lack of awareness, action, maintenance, and abandonment.

The perception of the benefits of being tested was lower at the stage of decision not to act than at all of the other stages.

The instrumental component of the attitude (considering being tested as useful and beneficial) was higher at the stages of action, maintenance, and abandonment than at both the stages of lack of awareness and decision not to act. Moreover, it was higher at the stage of decision to act than at the stage of decision not to act.

Finally, the emotional component of the attitude (considering being tested as pleasant and relaxing) was higher at the stage of action than at both the stages of lack of awareness and decision not to act. Furthermore, it was higher at the stage of maintenance than at the stage of decision not to act.

We also found differences among stages in diverse sociodemographic variables, such as age (*p* = .000), nationality (*p* = .000), level of studies (*p* = .000), socioeconomic status (*p* = .007), and employment situation (*p* = .000). There were no significant differences either in sex or habitat quota.

The group of people at the stage of decision not to act was characterised by being older, having less unemployment, and more retired people or pensioners than the other stages. The stage of decision to act included more young people, more students, fewer people of Spanish nationality, and more Latin Americans and people from other European countries. At the stages of action and maintenance, the percentage of people with university studies was higher than at the other stages, and they also had a somewhat higher socioeconomic status (Table 2).

## Discussion

The objective of this study was to analyse, in a representative sample of the Spanish population, the psychosocial determinants of late diagnosis of HIV contemplated in various models of health as a function of the population's stage of change concerning HIV-testing.

Firstly, the results showed that approximately two thirds of the population had never been tested. Most of the 27% who had taken the HIV test had done so occasionally. The predominant stage was lack of awareness. The most frequent barriers were related to the health system and to stigma. Both barriers had been reported in the literature [33].

Secondly, this study has shown the association of several of the psychosocial determinants analysed in the decision to be tested for HIV and also that such determinants differed depending on the respondents' stage of change.

On the one hand, results suggested the positive association in HIV-testing of the perception of risk, perceived self-efficacy, proximity to people who had been tested, perceived benefits of knowing the diagnosis, and a positive instrumental and emotional attitude. However, proximity to people who had been tested was the variable with the largest size of differences, thus confirming the important role of subjective norms on attitudes and behavior [14, 15]. Results of studies of other health behaviors had shown that the perceived benefits and self-efficacy increased across the stages, whereas the perception of threat, like the perception of susceptibility, was lower at the stages prior to the decision stage [18].

Nevertheless, the results also revealed psychosocial determinants presenting a negative association with the adoption of this health behavior. In this sense, it was observed that the underestimation of HIV risk, stereotypes about the infection, and the perceived severity of HIV were associated with the decision not to be tested. These results are consistent with the analysed theoretical framework, as individual susceptibility and perceived severity of the disease are important determinants of the probability of performing a health behavior [14]. Moreover, the literature has shown the relationship between low perception of risk and unrealistic optimism [16, 34]. There are two factors associated with such thinking that one is less likely than the average person to suffer undesirable events. On the one hand, the more severe a disease is perceived, the stronger the personal conviction that their own likelihood of contracting the disease is lower than that of a similar person. On the other hand, people holding stereotypes about the kind of person who acquires a certain disease may use such stereotypes as a mechanism to defend their identity. If the type of person who can contract the disease is highly stereotyped, an ego-defensive role may compel them to not consider themselves as representative of that kind of person [34]. As a consequence, they rarely consider themselves to be representative of that prototype.

It is also important to note that research on late HIV diagnosis had produced conflicting results about the role played by the perception of risk of infection. Thus, whereas some argue that low perception of risk is a barrier to early diagnosis [34], others have not found this relationship or have even found that perception of risk decreased the probability of the intention to be tested translating into action [35–37]. The results of this study may shed some light on this controversy, indicating that maybe the combination of the above-mentioned determinants and their evolution across the stages of change can lead to positive results.

This study has a number of limitations. Firstly, those arising from its correlational nature do not allow the establishment of causal conclusions.

Secondly, we used few indicators per variable to facilitate the surveyed population's responding to a telephone interview that included an important set of variables. This limitation could cause the measured constructs to be under-represented and is therefore a threat to validity. However, the method used to design the survey provides evidence of content validity, as the items were drafted based on the literature review and a prior qualitative study. Likewise, the psychometric recommendations for the elaboration of the items were rigorously followed. Furthermore, the relationships found were consistent with the predictions of the theoretical frameworks used. In addition, the restriction of items allowed us to analyse the most relevant psychosocial determinants included in models of health as a whole in a representative sample of the Spanish population. These issues could serve to alleviate this limitation, although it will be necessary for future research to examine this in depth and confirm the findings.

Another limitation of this study may be derived from the rate of non-respondents. This limitation is usual in studies employing surveys. In the present study, this rate was within the normal range for this kind of study. However, this may have implications because certain sociodemographic characteristics (higher educational level and socioeconomic status, women, single, etc.) may be different from those in the general population. Because of the design of the present study, sex quota and age were similar to those of the general population in Spain over 16 years old [38]. However, the percentage of people who had higher studies was larger than the datum reported by the Instituto Nacional de Estadistica [39]. Higher educational level could be related to several variables associated with HIV-testing (degree of HIV knowledge, beliefs, attitudes, etc.). Hence, the data provided should be interpreted in the light of this limitation.

Moreover, the survey did not collect certain important variables such as sexual behavior or HIV status. It was decided not to ask these questions to limit the refusal to respond that such personal questions could produce in a phone survey. Nevertheless, in order to control that the perception of risk of infection was associated with erroneous knowledge about the transmission of HIV, the survey contained a question on the subject. No significant differences were found among the stages in this item.

Finally, it is important to note that, in Spain, the strategy of testing followed is opt-in, that is, doctors offer the HIV test based on indicators or suggestive symptomatology. The fact that the testing is sometimes provider-initiated does not invalidate the results of this study. The stage of change and associated beliefs could influence acceptance or rejection of the test when it is offered by the doctor. Moreover, opportunities of missed diagnosis [33] in primary care are documented and therefore, it would also be useful for the people themselves to request the test from their doctor. Thus, despite the diagnosis strategy used, expanding our knowledge about the determinants and stages of change could be useful to increase early diagnosis.

## Conclusions

Studies of other health behaviors had determined the usefulness of grouping people into psychologically relevant stages and studying the differentiation of these stages [7, 20]. However, to our knowledge, this not had been studied in the specific case of HIV-testing. The results of this study show that there are different sociocognitive and motivational profiles depending on people's decision stage concerning HIV-testing. Knowing this profile may allow us to design interventions to influence the psychosocial determinants that characterise each stage of change.

## Additional file

Additional file 1: “Telephone interview questionnaire” contains the translation into English of the questionnaire used in the telephone interviews. (DOC 201 kb)

## Abbreviations

CATI: Computer-assisted telephone interviewing
HBM: Health belief model
MPAP: Model of the precaution adoption process
PMT: Protection motivation theory
SCT: Social cognitive theory
TPB: Theory of planned behavior
TRA: Theory of reasoned action

## Footnotes

1 The Random Dialing system uses the information obtained from the Telecommunications Market Commission. From this information, a series of telephone numbers (landline and mobiles) are extracted in proportion to the interviews to be carried out in each geographic sphere. These are the seed numbers. From them, the system extracts other numbers above and below them, as a function of a randomly determined sequence. As the geolocalisation of mobile phones cannot be determined a priori, it first obtains the percentage of the sample of homes that only have a mobile phone. Then the CATI system completed the remaining missing goals with landline phones. The system thus designed generates the same probability for all the homes to be included in the sample.
