# Supplementary material for: Psychosocial determinants of HIV testing across stages of change in Spanish population: a cross-sectional national survey
Source: BMC Public Health. 2017 Mar 7;17:234. doi: 10.1186/s12889-017-4148-4 (PMC5341365; doi:10.1186/s12889-017-4148-4)
Supplement: Additional file 1: — “Telephone interview questionnaire” contains the translation into English of the questionnaire used in the telephone interviews. (DOC 201 kb) [file 12889_2017_4148_MOESM1_ESM.doc]

**Additional file 1: Telephone interview questionnaire**

**PRESENTATION**

| *Good day/afternoon. My name is (name of the interviewer) and I am an interviewer from Opinatica an Institute that performs studies of public opinion. We are doing a survey on various topics of citizens’ interest and have randomly selected this home to perform an interview. It will be take a few minutes. Of course, your responses are completely anonymous.* |
| --- |

***CONTACT***

Q1. To establish what questions to ask, first, we need to know a few things.

Firstly, do you mind telling me your age?

| *Less than 16* | *(END OF CONTACT)* | | |
| --- | --- | --- | --- |
|  |  |  |  |
| *Age* |  |  |  |

| *Age in groups* | |
| --- | --- |
| *16 to 29* | *1* |
| *30 to 44* | *2* |
| *45 to 64* | *3* |
| *65 and over* | *4* |

Q2. And also, do you mind telling me your nationality?

| *Spanish* | *1* |
| --- | --- |
| *European Union Country* | *2* |
| *Other European country* | *3* |
| *Latin American* | *4* |
| *North American* | *5* |
| *African country* | *6* |
| *Other countries* | *7* |
| *No reply* | *9* |

Q3. Sex (DO NOT ASK)

| *Man* | *1* |
| --- | --- |
| *Woman* | *2* |

***INTERVIEW***

Q4. Well, in your case we would have to ask you some things, specifically... about AIDS: do you know what AIDS is, what we mean when we talk about AIDS?

| *Acquired immunodeficiency syndrome, a disease, epidemic or something like that…* | *1* |
| --- | --- |
| *A clearly incorrect answer* | *2* |
| *(WRITE DOWN LITERALLY ONLY FOR FIRST SUPERVISION)* | |
| *Doesn't know/No reply* | *9* |

| *(2 AND 9 -> END OF THE INTERVIEW)* |
| --- |

Q5. And do you have any idea of what we mean when we refer to HIV?

| Human immunodeficiency virus, something related to AIDS, a disease, epidemic or something like that… | 1 |
| --- | --- |
| An incorrect answer | 2 |
| *(WRITE DOWN LITERALLY)* | |
| Doesn't know/No reply | 9 |

Q6. Actually, HIV (Human Immunodeficiency Virus) is the determining infectious agent of the Acquired Immunodeficiency Syndrome, of AIDS.

We're going to ask some questions about AIDS, but only about your attitudes and opinions of AIDS. Of course, we will never ask you whether are sick or infected by HIV. For example, what do you think about this statement?: “AIDS is a deadly disease”

*(READ RESPONSE OPTIONS)*

| Totally agree | 4 |
| --- | --- |
| Agree somewhat | 3 |
| Slightly agree | 2 |
| Strongly disagree | 1 |
| Doesn't know/No reply | 9 |

Q7. And this one?: “AIDS is a very serious disease”

| Totally agree | 4 |
| --- | --- |
| Agree somewhat | 3 |
| Slightly agree | 2 |
| Strongly disagree | 1 |
| Doesn't know/No reply | 9 |

Q8. A last question: "In society, there is a lot of rejection towards people with HIV”

*(REREAD THE SCALE, IF NECESSARY)*

| Totally agree | 4 |
| --- | --- |
| Agree somewhat | 3 |
| Slightly agree | 2 |
| Strongly disagree | 1 |
| Doesn't know/No reply | 9 |

Q9. According to our way of life and our habits, almost everyone has some risk of contracting AIDS. Thinking about your way of life and your habits, to what extent do you think that you could become infected with HIV?

| Very likely | 4 |
| --- | --- |
| Fairly likely | 3 |
| Unlikely | 2 |
| Not at all likely | 1 |
| Doesn't know/No reply | 9 |

Q10. Now I'm going to say a series of phrases for you to tell me the extent to which you agree with each one of them.

“You think you are less likely to become infected by HIV than the average of the population”

*(AS A RULE, READ RESPONSE OPTIONS)*

| Totally agree | 4 |
| --- | --- |
| Agree somewhat | 3 |
| Slightly agree | 2 |
| Strongly disagree | 1 |
| Doesn't know/No reply | 9 |

Q11. “You don't think that it can happen to you because it is an infection that only people with other lifestyles different from yours can get.”

| Totally agree | 4 |
| --- | --- |
| Agree somewhat | 3 |
| Slightly agree | 2 |
| Strongly disagree | 1 |
| Doesn't know/No reply | 9 |

Q12. Another phrase: “You would be afraid to discover that you have HIV”

| Totally agree | 4 |
| --- | --- |
| Agree somewhat | 3 |
| Slightly agree | 2 |
| Strongly disagree | 1 |
| Doesn't know/No reply | 9 |

Q13. Could you tell me the extent to which you feel capable of going to get tested for HIV? We are not asking whether you have gotten tested or are going to get tested, only to what degree you feel capable of doing it.

| Very capable | 4 |
| --- | --- |
| Fairly capable | 3 |
| Not very capable | 2 |
| Not at all capable | 1 |
| Doesn't know/No reply | 9 |

Q14. Imagine that you did the test and that it came out positive, to what extent do you think you would feel capable of facing that outcome, that situation?

| Very capable | 4 |
| --- | --- |
| Fairly capable | 3 |
| Not very capable | 2 |
| Not at all capable | 1 |
| Doesn't know/No reply | 9 |

Q15. Have any people close to you been tested for HIV?

| Many | 4 |
| --- | --- |
| Quite a lot | 3 |
| Few | 2 |
| None | 1 |
| Doesn't know/No reply | 9 |

Q16. On a scale of 0 to 10, tell me, for you, to what extent do you consider that taking the HIV test and knowing its outcome would be:

Harmful or beneficial? (scores approaching 0 mean that it is deemed harmful and approaching 10 means it is considered beneficial)

|  |  | Score  between  0 and 10 |  | Does not know/  No reply |
| --- | --- | --- | --- | --- |
| Q16 | Harmful |  | Beneficial | 99 |
| Q17 | Useless |  | Useful | 99 |
| Q18 | Unpleasant |  | Pleasant | 99 |
| Q19 | Stressful |  | Relaxing | 99 |

Q17. Useless or useful?

*(USE SCALE FROM 0 TO 10)*

Q18. Unpleasant or pleasant?

*(USE SCALE FROM 0 TO 10)*

Q19. Stressful or relaxing?

*(USE SCALE FROM 0 TO 10)*

Q20. To what extent do you think that a person can become infected by HIV through sexual intercourse if condoms are not used?

| Very likely | 4 |
| --- | --- |
| Fairly likely | 3 |
| Unlikely | 2 |
| Not at all likely | 1 |
| Doesn't know/No reply | 9 |

Q21. Remember: we are not asking whether or not you have AIDS, but tell me the extent to which you agree with each of these statements: “If you had HIV, you think it would be positive to know it so you could receive treatment and control the disease as soon as possible".

*(INSIST: WE DO NOT WANT TO KNOW WHETHER OR NOT THE PERSON HAS HIV, BUT TO IMAGINE THAT SITUATION)*

| Totally agree | 4 |
| --- | --- |
| Agree somewhat | 3 |
| Slightly agree | 2 |
| Strongly disagree | 1 |
| Doesn't know/No reply | 9 |

Q22. “It would be good to get the test because, in spite of the fact that you could have HIV, having exact information would relieve you about your health status”.

| Totally agree | 4 |
| --- | --- |
| Agree somewhat | 3 |
| Slightly agree | 2 |
| Strongly disagree | 1 |
| Doesn't know/No reply | 9 |

Q23. “If you had HIV, it would be good to know it as soon as possible because that would help to protect and prevent the transmission to your partner or partners”.

| Totally agree | 4 |
| --- | --- |
| Agree somewhat | 3 |
| Slightly agree | 2 |
| Strongly disagree | 1 |
| Doesn't know/No reply | 9 |

Q24. “If people who are important for you wanted you to get tested for HIV, you would do it”.

| Totally agree | 4 |
| --- | --- |
| Agree somewhat | 3 |
| Slightly agree | 2 |
| Strongly disagree | 1 |
| Doesn't know/No reply | 9 |

Q25. Have you ever been tested for HIV? (MAKE IT CLEAR THAT WE ARE NOT GOING TO ASK ABOUT THE OUTCOME IF HE/SHE ANSWERS YES). Just tell me your position from among the following ones:

*(ONCE AGAIN: WE TO NOT WANT TO KNOW WHETHER OR NOT THE PERSON HAS HIV, ONLY IF THEY HAVE BEEN TESTED)*

| No, I have never thought about getting tested | 1 |
| --- | --- |
| No, and I'm not going to do it | 2 |
| No, but I intend to do it soon | 3 |
| Yes, I've sometime been tested for HIV | 4 |
| Yes, I usually get tested regularly | 5 |
| Yes, but I do not intend to get tested any more | 6 |
| No reply | 9 |

*For those who answer Yes (3, 4, 5 and 6) TO Q25*

Q26. Could you tell me the reasons why you got tested for HIV?

*(TO THOSE WHO COME FROM Q25.3 -> Could you tell me what are your reasons for getting tested for HIV?)*

*(MULTIPLE; READ; ROTATE)*

| It just seemed like a good idea | 1 |
| --- | --- |
| Your doctor or another health professional suggested that you get tested | 2 |
| You are worried about the possibility of being infected | 3 |
| Your partner or someone important for you has suggested getting tested or has asked whether you have been tested | 4 |
| You felt ill and decided to get a general checkup  *(TO THOSE COMING FROM Q25.3 -> You felt ill and have decided to get a general checkup)* | 5 |
| You had doubts about whether some partner of yours could have HIV or any other sexually transmitted infection | 6 |
| The doctor or nurse told you that you had been tested because it was just one more part of the routine checkup that they did  *(TO THOSE COMING FROM Q25.3 -> The doctor or nurse told you that you are going to be tested because it is just one more part of the routine checkup that they are going to do)* | 7 |
| Doesn't know/No reply *(INSIST ON A RESPONSE)* | 9 |

*To those who answer No (1, 2, and 3) or that THEY WOULD NOT GET TESTED AGAIN (6) ON Q25*

Q27. Let us look at other statements and you indicate again the extent to which you agree with each one of them:

"You are concerned that someone might discover that you are going get tested for HIV”.

| Strongly agree | 4 |
| --- | --- |
| Pretty much agree | 3 |
| Slightly agree | 2 |
| Strongly disagree | 1 |
| Doesn't know/No reply | 9 |

Q28. “You are afraid that your name might appear on public records if you get tested for HIV”.

| Strongly agree | 4 |
| --- | --- |
| Pretty much agree | 3 |
| Slightly agree | 2 |
| Strongly disagree | 1 |
| Doesn't know/No reply | 9 |

Q29. “You are worried that who are people important to you will reject you if you get tested for HIV”.

| Strongly agree | 4 |
| --- | --- |
| Pretty much agree | 3 |
| Slightly agree | 2 |
| Strongly disagree | 1 |
| Doesn't know/No reply | 9 |

Q30. “You have no time to go and get tested for HIV”.

| Strongly agree | 4 |
| --- | --- |
| Pretty much agree | 3 |
| Slightly agree | 2 |
| Strongly disagree | 1 |
| Doesn't know/No reply | 9 |

Q31. “You don't know where to go to get tested for HIV”.

| Strongly agree | 4 |
| --- | --- |
| Pretty much agree | 3 |
| Slightly agree | 2 |
| Strongly disagree | 1 |
| Doesn't know/No reply | 9 |

*IF 4 OR 9 IN Q31, SCORE AS 9 IN Q32 AND GO ON TO Q33*

Q32. “The place where the HIV test is done is very far away”.

| Strongly agree | 4 |
| --- | --- |
| Pretty much agree | 3 |
| Slightly agree | 2 |
| Strongly disagree | 1 |
| Doesn't know/No reply | 9 |

Q33. “Your doctor did not recommend you to be tested for HIV”.

| Strongly agree | 4 |
| --- | --- |
| Pretty much agree | 3 |
| Slightly agree | 2 |
| Strongly disagree | 1 |
| Doesn't know/No reply | 9 |

Q34. “You would feel ashamed or embarrassed to talk with your doctor about the possibility of getting tested for HIV”.

| Strongly agree | 4 |
| --- | --- |
| Pretty much agree | 3 |
| Slightly agree | 2 |
| Strongly disagree | 1 |
| Doesn't know/No reply | 9 |

Q35. “You are concerned about what your doctor might think of you if you mention getting tested for HIV”.

| Strongly agree | 4 |
| --- | --- |
| Pretty much agree | 3 |
| Slightly agree | 2 |
| Strongly disagree | 1 |
| Doesn't know/No reply | 9 |

FOR EVERYONE

**CLASSIFICATION DATA**

Q36. To conclude, we need to ask you some questions to be able to classify your responses with those of other people.

Are you the main household provider, that is, the person who usually contributes more income?

| Yes | 1 |
| --- | --- |
| No | 2 |

Q37. Could you tell me your attained educational level? (IF IN DOUBT, WRITE DOWN EXACTLY WHAT THE INTERVIEWEE SAYS)

| Less than primary studies | 1 |
| --- | --- |
| Primary studies | 2 |
| Secondary studies | 3 |
| Middle university studies | 4 |
| Higher university studies | 5 |
| Doesn't know/No reply | 6 |
| *(WRITE DOWN)* | |

Q38. Could you tell me your current occupation? (IF IN DOUBT, WRITE DOWN EXACTLY WHAT THE INTERVIEWEE SAYS)

| Entrepreneur with > 6 employees | 1 |
| --- | --- |
| Entrepreneur with < 6 employees | 2 |
| Farmer without employees | 3 |
| Liberal profession/autonomous | 4 |
| Directors and higher managers | 5 |
| Industrial technicians and specialists | 6 |
| Office technicians and specialists in services | 7 |
| Out-of-office technicians and specialists in services | 8 |
| Civil servants | 9 |
| Day laborers and subordinates | 10 |
| Unemployed | 11 |
| Retirees and pensioners | 12 |
| Rest of workers | 13 |
| Home-keepers | 14 |
| Students | 15 |
| *(WRITE DOWN)* | |

| **We have finished. Thank you very much** |
| --- |
